# Campylobacter at the Human–Food Interface: The African Perspective

**DOI:** 10.3390/pathogens8020087

**Published:** 2019-06-25

**Authors:** Nikki Asuming-Bediako, Angela Parry-Hanson Kunadu, Sam Abraham, Ihab Habib

**Affiliations:** 1 School of Veterinary Medicine, College of Science, Health, Education and Engineering, Murdoch University, Perth 6150, Australia; nikkiabed@yahoo.com (N.A.-B.); s.abraham@murdoch.edu.au (S.A.); 2 CSIR-Animal Research Institute, Achimota P.O. Box AH20, Ghana; 3 Department of Nutrition and Food Science, University of Ghana, Legon P.O. Box LG134, Ghana; aparry-hanson@ug.edu.gh; 4 Veterinary Medicine Department, College of Food and Agriculture, United Arab of Emirates University, Al Ain P.O. Box 1555, UAE

**Keywords:** campylobacteriosis, developing countries, one health, zoonoses, antimicrobial resistance

## Abstract

The foodborne pathogen *Campylobacter* is a major cause of human gastroenteritis, accounting for an estimated annual 96 million cases worldwide. Assessment of the true burden of *Campylobacter* in the African context is handicapped by the under-reporting of diarrhoeal incidents and ineffective monitoring and surveillance programmes of foodborne illnesses, as well as the minimal attention given to *Campylobacter* as a causative agent of diarrhoea. The present review of the literature highlights the variability in the reported occurrence of *Campylobacter* in humans and animal food sources across different countries and regions in Africa. *Campylobacter* infection is particularly prevalent in the paediatric population and has been isolated from farm animals, particularly poultry, and foods of animal origin. The reported prevalence of *Campylobacter* in children under the age of five years ranges from 2% in Sudan to 21% in South Africa. In poultry, the prevalence ranges from 14.4% in Ghana to 96% in Algeria. This review also highlights the alarming trend of increased *Campylobacter* resistance to clinically important antimicrobials, such as ciprofloxacin and erythromycin, in humans and food animals in Africa. This review adds to our understanding of the global epidemiology of *Campylobacter* at the human–food animal interface, with an emphasis from the African perspective. Interinstitutional and intersectoral collaborations, as well as the adoption of the One Health approach, would be useful in bridging the gaps in the epidemiological knowledge of *Campylobacter* in Africa.

## 1. Introduction

*Campylobacter* is a gram-negative, non-spore forming, curved or spiral bacilli, which are oxygen sensitive and prefer to grow under micro-aerobic conditions [1,2,3]. Some *Campylobacter* species are thermotolerant; for instance, *Campylobacter jejuni* (*C. jejuni*) and *Campylobacter coli* (*C. coli*), which are of critical importance to food safety, grow optimally at 42 °C [2]. In humans, *C. jejuni* and *C. coli* are the main culprits of campylobacteriosis, a very widely recognised enteric illness that can be transmitted to humans through the consumption of undercooked meat, especially poultry, contaminated water and milk, and contact with farm animals such as poultry and livestock [4,5,6,7]. It has been widely accepted that improper handling and consumption of contaminated food (notably poultry meat) accounts for the majority of human cases [8,9]. Campylobacteriosis in humans is characterised by watery and/or bloody diarrhoea, abdominal pain, cramps, fever, malaise, and vomiting [10,11,12]. This is especially dangerous for young children who are more prone to dehydration and loss of nutrients, such as sodium and protein, as a consequence of the diarrhoeal illness [13].

The pathogenesis of *Campylobacter* infection is hypothesised to several mechanisms, however it is not yet fully understood. It has been shown that the expression of genes involved in motility, colonization, epithelial cell invasion, and toxin production play an important role in the disease development [14,15]. Several genes (i.e., *fla*A and *flh*A), are essential for the mobility/passage of *Campylobacter* through the stomach and gut environment [16]. In addition, several proteins (encoded by the *cad*F, *doc*A, *rac*R, *vir*B11, *cia*B, and *iam* genes) on the surface of *Campylobacters* have been shown to promote the adherence and invasion of epithelial cells of the intestine [15,17,18]. *Campylobacter has* also been found to excrete several cytotoxins (encoded by the *cdt*A, *cdt*B, *cdt*C, and *wla*N genes) that contribute to the development of human illness [19,20]. Moreover, *C. jejuni* is able to produce superoxide, dismutase enzyme (encoded by the *sod*B marker), which catalyses the breakdown of superoxide radicals and hence play a major role in defending *Campylobacters* from oxidative damage [21].

In low and middle-income countries, the true incidence of *Campylobacter* is difficult to determine since there are limited systematic surveillance efforts to detect outbreaks and provide isolates that could be used for source-attribution and risk assessment [22]. The infection with *Campylobacter* is regarded as hyper-endemic in many developing countries, due to poor food and environmental sanitation, and close contact with animals at domestic settings in rural and agricultural communities, among many other factors [23]. *Campylobacter* is one of the most frequently isolated bacterial pathogens from the stools of infants with diarrhoea in several developing countries [3,24]. According to the World Health Organisation [9], 40% of the global foodborne disease burden is inflected on children under the age of five years, with the highest burden per population observed in Africa.

Although antimicrobial therapy is not generally indicated in most campylobacteriosis cases, treatment can decrease the duration and reduces the symptoms if it is initiated early in severe cases that warrant antimicrobial intervention [25]. Macrolides (specifically erythromycin) and fluoroquinolones (specifically ciprofloxacin) are considered as the first- and second-choice of antimicrobials, respectively, for the treatment of severe human *Campylobacter* infections [9]. It has been claimed that the spread of antibiotic resistant bacteria/genes to humans through the food chain could be promoted by the uncontrolled extensive use of antibiotics for prophylaxis and treatment in the primary animal production [26]. The situation of antimicrobial resistance in *Campylobacter* is not fully understood across the African continent, despite some reports indicating varying trends at the human-food animal interface [5,25,27,28,29].

This review collates the knowledge on the epidemiology of *Campylobacter* in humans and food animals in Africa, antimicrobial resistance patterns, and suggestions for management. Specifically, this review aims at elucidating—(i) the prevalence of *Campylobacter* in humans, particularly children, across different regions of the African continent; (ii) the prevalence of *Campylobacter* in foods of animal origin; and (iii) *Campylobacter*’s resistance to antimicrobial agents, notably macrolides and fluoroquinolones.

## 2. Prevalence of *Campylobacter* in Humans

To facilitate data consolidation and regional comparisons, in this review we collate evidence from literatures based on categorisation of the African continent into five geographical sub-regions (East Africa, Central Africa, West Africa, Southern Africa, and North Africa), in accordance with the United Nations Geoscheme for Africa [30]. Table 1 provides a summary of the published research depicting *Campylobacter* prevalence rates in humans.

### 2.1. East Africa

In East Africa, *Campylobacter* infections have been recorded in both rural and urban areas, particularly among children. The prevalence varies between countries (see Table 1), with the highest reported rate being 21% in diarrhoeal children in Malawi [61]. A cross-sectional study conducted from July to October 2012 in the south-western town of Jimma, Ethiopia, detected the presence of *Campylobacter* in the stools of 16.7% of 227 diarrhoeal children under the age of five years [5]. Another study conducted between October 2011 and March 2012 found a prevalence of 15.4% in 285 diarrhoeal children undergoing treatment at the University of Gondar Hospital in northwest Ethiopia [31]. Both studies found that the frequency of *Campylobacter* was higher in malnourished children and in those from households that lacked a source of clean water and had direct contact with domestic animals, particularly hens. Interestingly, the education level of the caregiver, family size, or handwashing before preparing food or eating or after defecation showed no statistically significant association with a positive culture of *Campylobacter*.

In a case-control study conducted in Kisii in south-western Kenya, analysis of 312 stool samples (156 cases and 156 controls) identified the presence of *Campylobacter* in 5.8% and 1% of cases and controls, respectively [33]. In Madagascar, 9.7% of 2196 diarrhoeal stool samples collected from 14 districts during the 2008–2009 rainy season contained *Campylobacter* spp. [34]. In the southern Malawian city of Blantyre, 1941 faecal samples were collected between 1997 and 2007 from children hospitalised with diarrhoea—analysis of these samples indicated that 21% of the samples contained *Campylobacter* compared with 14% of the samples from 507 non-diarrhoeic children [35]. An analysis of 529 stool samples collected from diarrhoeal children at the Manhiça District Hospital in southern Mozambique showed a low *Campylobacter* presence of 1.7% [36].

In Morogoro in eastern Tanzania, Komba, Mdegela, Msoffe, Nielsen and Ingmer [37] detected *Campylobacter* in 11.4% of the stool samples taken from 1195 individuals. The prevalence among symptomatic and young individuals was higher than in asymptomatic and adult individuals. In the northern Tanzanian city of Mwanza, a cross-sectional study of 300 children with acute watery diarrhoea in two hospitals revealed that 9.7% of the stool samples tested positive for *Campylobacter* [38]. In another cross-sectional study in rural and urban areas of Morogoro in eastern Tanzania from January 2003 to December 2004, the prevalence of *Campylobacter* was reported as 9.3% in 632 human stool samples, with *C. jejuni* accounting for more than 90% of the positive isolates [39].

Similar to the studies from Tanzania, Mshana, Joloba, Kakooza and Kaddu-Mulindwa [27] recorded a 9.3% isolation rate of *Campylobacter* in 226 stool samples from diarrhoeal children attending the Mulago Hospital in Kampala, the capital city of Uganda, with *C. jejuni* being the most frequently detected species (80.9%). Mshana et al. further reported a higher infection rate of 10.9% in children under two years of age compared with a rate of 8.5% in children over two years of age. The researchers proposed that the increase in protective antibodies with age may account for the decreased rate of infection in older children.

### 2.2. Central Africa

There are limited published studies from the central African region. An analysis of 194 stool samples from 98 children with acute diarrhoea and 96 children without diarrhoea under the age of five in Angola’s capital city of Luanda found that *Campylobacter* was present in 15% of the samples overall, with 23% present in the stools of diarrhoeic children compared with 6% in the stools of non-diarrhoeic children [40]. Multiplex real-time polymerase chain reaction (mPCR) was used to analyse the samples. Other pathogens, including *Escherichia coli*, *Salmonella*, *Cryptosporidium,* and *Shigella*, were detected in all the samples, regardless of diarrhoeal status.

### 2.3. West Africa

In Ouagadougou, the capital of Burkina Faso, Sangaré, Nikiéma, Zimmermann, Sanou, Congo-Ouédraogo, Diabaté, Diandé and Guissou [41] collected stool samples from 1246 enteritis patients from 2006 to 2008 and reported a *Campylobacter* isolation rate of 2.3%, with *C. jejuni* accounting for 51.8%, *C. coli* accounting for 13.8%, and *C. upsaliensis* accounting for 3.5%. In Kumasi, the capital of Ghana, Karikari, Obiri-Danso, Frimpong and Krogfelt [42] reported a *Campylobacter* prevalence of 17.3% in 202 patients who visited the Komfo Anokye Teaching Hospital from May to August 2013.

In Liberia, researchers reported a noteworthy *Campylobacter* isolation rate of 44.9% in 341 children from a crowded urban slum compared with only 28% in 518 children from a cleaner rural area. [43]. The children were aged between six months and five years. The authors found that the prevalence of *Campylobacter* increased in children aged over 18 months, arguing that this may be attributed to increased contact with animals and the environment. Other risk factors for infection were the post-weaning consumption of contaminated food and water.

In Nigeria, Africa’s most populous country, analysis of 292 stool samples taken from people in hospitals across four agricultural zones in the north-western state of Sokoto detected the presence of *Campylobacter* in 55% of the samples [44]. Another study in the same state found that 70% of 23 pregnant women and 43% of 57 non-pregnant women were stool-positive for *Campylobacter* [45]. In this study, exposure was attributed to poor environmental conditions in the homes of patients [45]. In Enugu, in the south-eastern state of Nigeria, a lower isolation rate of 8.3% was found in 514 children under the age of five years, with *C. jejuni* accounting for 93% of the positive isolates [46]. Similarly, Samuel, Aboderin, Akanbi II, Adegboro, Smith and Coker [23] detected *Campylobacter* in 8.2% of the stool samples from 306 diarrhoeal children in Ilorin in the middle belt zone of Nigeria, with all positive isolates being found in children under the age of two years. The authors indicated that the key risk factors were exposure to an unclean environment and the consumption of contaminated foods and water after weaning [23].

### 2.4. Southern Africa

In the Venda region, located in the northern part of South Africa, *C. jejuni* was detected in 10.2% of 322 stool samples collected from patients admitted to a hospital [47]. Although other pathogens such as *H. pylori*, *Arcobacter butzleri*, *A. skirowii,* and *A. cryaerophilus* were also present, *C. jejuni* was significantly associated with diarrhoea. In another study in South Africa, samples of diarrhoeal stools were taken from 565 people in rural areas in the northernmost district of Vhembe, and analysis using the filtration method detected *Campylobacter* in 20.3% of the samples. A higher isolation rate of 30.4% was seen in the samples taken from children under the age of two [48]. Lastovica [49] analysed 5443 diarrhoeal stool samples collected from the Red Cross Children’s Hospital in the coastal city of Cape Town between 1990 and 2005 and found that 40% contained *C. jejuni,* 24.6% contained *C*. *concisus,* and 23.6% contained *C. upsaliensis.* The isolation was carried out using the Cape Town Protocol, which may have contributed to the high levels detected. In the rural Limpopo province of north-eastern South Africa, *Campylobacter* was isolated from 20% of the stool samples taken from 60 HIV-positive individuals with chronic diarrhoea [50]. Forty of the 60 individuals tested were positive for other diarrhoeal agents, including *E. coli*, *Shigella*, *Salmonella*, *Plesiomonas shigelloides,* and *Aeromonas* spp. HIV infection is known to seriously compromise immunity; hence, patients were susceptible to a wide range of infections. Further, in two interrelated studies undertaken in a Durban hospital, *Campylobacter* was found in 21% of the stool samples taken from 126 malnourished inpatient children compared with 7% of the stool samples taken from 352 randomly selected outpatient children [51]. Other pathogens such as *Salmonella*, *E. coli,* and *Shigella* were isolated from both groups. Malnutrition compromises the body’s defence system, thus increasing susceptibility to infection.

### 2.5. North Africa

A study by Abushahba [52] in Assiut in Egypt, located about 375 km south of the capital Cairo, found that 27.5% of 80 human stool samples screened positive for *Campylobacter*. Of the participants in that study, 33 were infants under the age of 12 months. The key risk factors for infection were impaired immunity and residential conditions in villages, with poor hygiene and poultry rearing in households. In a prospective study conducted in Abu Homos, an agricultural community in northern Egypt, from 1995 to 2003, *Campylobacter* was isolated from 9.37% of 6562 faecal samples collected from 1057 children [53]. In the Gharbia Governorate located in the Nile Delta region of Egypt, El-Tras, Holt, Tayel and El-Kady [54] found a prevalence of 12.3% and 2.8% for *C. jejuni* and *C. coli*, respectively, in 106 rural children from households that owned poultry. Poultry is a major reservoir for *Campylobacter* and is, therefore, an important source of transmission—backyards or coops with wet litter and poor sanitation increase the risk of human exposure. In Zagazig in the eastern part of the Nile Delta, Awadallah, Ahmed, El-Gedawy and Saad [56] detected a *Campylobacter* prevalence of 2.7% in 110 stool samples sourced from the El-Ahrar General Hospital from September 2012 to April 2014. Hassanain [57] reported a higher *Campylobacter* prevalence of 16.66% in 48 human faecal samples collected from individuals in contact with food-producing animals in the Giza Governorate in central Egypt.

From 1995 to 1998, a case-control study of 397 children under the age of three years in the Abu Homos region of northern Egypt reported 3477 episodes of diarrhoea of which 366 (10.5%) were associated with *Campylobacter* [58]. The presence of animals in the house, particularly in cooking areas, and other unhygienic conditions were major risk factors for infection. Breastfeeding did not appear to reduce the risk of *Campylobacter*-associated diarrhoea, but a reduced risk was associated with adequate toilet facilities. At Abbassia Fever Hospital in Cairo, 869 *Salmonella*, *Shigella,* and *Campylobacter* strains were isolated from 6278 patients who visited the hospital from January 1986 to December 1993. Although *Salmonella* was the predominant strain at 53.5%, *Campylobacter* showed a prevalence of 16.8%, with *C. jejuni* present in 92 of the 146 *Campylobacter*-positive isolates [59]. 

In Sudan, an isolation rate of 2% was found in 437 stool samples of diarrhoeal children collected from January to December 2013 in suburban Khartoum [60]. The samples were also colonised by other pathogens, including *E. coli*, rotavirus A, *Shigella*, *Salmonella,* and *Giardia intestinalis*. Bacterial agents were the most common cause of diarrhoea and children over two years of age were frequently affected. Contaminated hands are a common source of foodborne infections and, given that proper handwashing is a challenge for this age group, this may have been a reason for the increased prevalence of diarrhoea [60].

## 3. Prevalence of *Campylobacter* in Foods of Animal Origin

Meat, eggs, milk, and other products from animals represent an important part of the diet of Africans [62]. On the African continent, varying rates of *Campylobacter* prevalence in food of animal origin have been reported ranging from 2% in beef to 90% in chicken carcasses. Table 2 provides a summary of the *Campylobacter* prevalence in foods of animal origin.

### 3.1. East Africa

Sheep and goat meat are an important part of the Ethiopian diet. A cross-sectional study of 398 sheep and goat carcasses from a private export abattoir in Debre-Zeit, 45 km south-east of Addis Ababa, between October 2007 and March 2008 found that *Campylobacter* was present in 10.1% of the samples, with *C. jejuni* accounting for 72% of the isolates [63]. The highest bacterial isolation was from the breast region of the carcasses, resulting from cross-contamination from intestinal contents during manual skinning, evisceration, processing, and contact with processors’ hands or knives. Additionally, washing of carcasses carries microorganisms from other parts of the body to the breast region. Therefore, effective hygienic practices and attentive evisceration during slaughter and dressing are important measures to reduce contamination.

In Kenya, Osano and Arimi [64] analysed 100 chicken and 50 beef samples from butchers, supermarkets, and markets in Nairobi and reported the presence of *Campylobacter* in 77% and 2% of chicken and beef samples, respectively. *C. jejuni* was the dominant species isolated from chicken, emphasising its potential role in zoonotic transmission between humans and poultry. In Tanzania, Kashoma, Kassem, John, Kessy, Gebreyes, Kazwala and Rajashekara [65] reported the presence of *Campylobacter* in 9.5% of 253 beef carcasses and 13.4% of 284 unpasteurised raw milk samples using mPCR. This was conducted from April 2013 to March 2014 and samples were sourced from Arusha, Iringa and Morogoro in Tanzania. The milk was obtained from milk vendors and milk tanks at milk collecting centres, while the beef swabs were taken from dressed carcasses. This study illustrates that the consumption of raw milk is a route for the transmission of *Campylobacter.* Cattle also carry *C. jejuni* and cross-contamination could occur during slaughter and milking. Therefore, slaughter and milking should be carried out using hygienic methods to minimise the transmission of pathogens to meat and milk and, consequently, to humans. Similarly, *Campylobacter* was detected in 9.3% of 107 cattle carcasses sampled from an abattoir in Morogoro in east Tanzania, while in meat shops, it was detected in 1.7% of samples [67]. The authors reported that the dressing and sale of meat was carried out in unhygienic environments and some shops sold chicken in addition to beef, thereby enhancing the risk of transfer.

### 3.2. Central Africa

In Cameroon, Nzouankeu, Ngandjio, Ejenguele, Njine and Wouafo [68] reported that 90% of 150 retail chickens obtained from eight markets in the capital Yaounde from February 2006 to January 2007 contained *Campylobacter* spp.. *E. coli* and *Salmonella* were also isolated from the samples using culture-based methods. *Campylobacter* is commensal in poultry, its primary host, and the risk of cross-contamination to the carcass during slaughter and processing is high if this is not carried out carefully and hygienically. Nzouankeu et al. further suggested the need to monitor poultry for pathogens and to minimise cross-contamination.

In a study of retail goat meat outlets in Lubumbashi in the Democratic Republic of the Congo, *Campylobacter* was found in 41.2% of 177 goat meat samples, 37.2% of 86 goat stomach samples, and 23.7% of 139 ready-to-eat goat skewers using polymerase chain reaction (PCR) [69]. The outlets comprised open air and semi-open-air markets, snack bars, and bars. The authors noted that the slaughter of animals was undertaken in unhygienic facilities and retail points, and no efforts were made to ward off flies or other vermin. However, cooking decreased the prevalence of *Campylobacter*, as evidenced by the lower rate recorded in the ready-to-eat goat skewers sourced from the same outlets. Thorough cooking of meat is an effective way of reducing the risk of infection in humans.

### 3.3. West Africa

In Ouagadougou, Burkina Faso, *Campylobacter* was detected in 50% of 20 poultry carcasses sourced from retail markets [70]. The carcasses were sold on tables in ambient temperatures without protection from dust or flies, the vendors did not wear gloves or aprons, and the retail environment was infested with lizards, rodents, and avian species. Kagambèga, Thibodeau, Trinetta, Soro, Sama, Bako, Bouda, Wereme N’Diaye, Fravalo and Barro [70] suggested that, although poultry is widely consumed in Burkina Faso, patients with diarrhoea are not routinely sampled for *Campylobacter*. However, in neighbouring Ghana, a lower isolation rate of 21.9% was detected in 132 poultry carcasses randomly selected from the Kejetia poultry slaughter unit in Kumasi [71]. *Campylobacter* is known to colonise the intestinal tract of poultry and cross-contamination can occur during slaughtering and processing if the carcass is not properly handled. Karikari, Obiri-Danso, Frimpong and Krogfelt [72] found that *Campylobacter* were present in 34.5% of beef, 23.9% of goat, 35.9% of sheep, and 36.3% of pig carcasses sampled from the Kumasi abattoir. The contamination of carcasses during manual skinning, evisceration, and processing at the abattoir were the reasons attributed to the prevalence rates recorded.

In Sokoto in north-western Nigeria, Salihu, Junaidu, Magaji and Rabiu [73] detected the presence of *Campylobacte*r in 4.8% of 146 raw milk samples from lactating herds between October 2007 and September 2008. All the positive samples contained *C. jejuni* biotype I. The authors suggested that animal and animal products were reservoirs for human infections. Analysis of breed distribution showed that White Fulani breeds had a higher prevalence (5.4%) than Sokoto Gudali breeds (4.7%) or Friesian–Sokoto Gudali crossbreeds (0.0%). Using culture methods, Salihu, Junaidu, Magaji, Abubakar, Adamu and Yakubu [74] detected *Campylobacter* in 81.9% of 681 chicken samples in Sokoto from November 2007 to October 2008. *C. jejuni* accounted for 60.9% of the isolates, followed by *C. coli* at 28%, and *C. lari* at 7%. Biotyping showed a prevalence of biotype I in *C. jejuni*, *C. coli,* and *C. lari*. *C. jejuni* I and *C. coli* I are found in humans; hence, the results were indicative of the zoonotic nature of the pathogen.

From January 2001 to May 2002, 300 fresh, refrigerated, or frozen chicken carcasses from retail outlets in Dakar in Senegal were analysed, with *Campylobacter* being present in 56% of the samples [75]. The contamination rates were found to be highest in the fresh samples at 76% and lowest in the frozen samples at 28%. Fifty-three percent of the refrigerated samples were also contaminated. *Campylobacter* is stress sensitive and freezing affects its viability, hence the lower rates found in frozen samples. Therefore, hygienic handling practices during slaughter and processing, and adequate personal hygiene and cooking techniques are important for infection control.

### 3.4. Southern Africa

In South Africa, van Nierop, Duse, Marais, Aithma, Thothobolo, Kassel, Stewart, Potgieter, Fernandes, Galpin and Bloomfield [76] found that *Campylobacter* was present in 32.3% of 99 fresh and frozen chicken carcasses sourced from retailers in Gauteng in the country’s north. Carcasses from supermarkets were more frequently contaminated with *Campylobacter* than those from butchers, which were more frequently contaminated with *Salmonella.* Similarly, in a study in Senegal, *Campylobacter* was isolated more frequently from fresh chicken than from frozen chicken. Culture methods detected an isolation rate of 32.3%, whereas PCR detected a rate of 43.4%. Detection methods play a key role in detecting prevalence, with PCR reported to be more sensitive in the detection of pathogens. However, the authors cautioned that the use of PCR may have resulted in false positives because of DNA contamination, the presence of inhibitory substances in the enrichment broths or the identification of non-viable pathogens as viable.

### 3.5. North Africa

In Algeria, Bouhamed, Bouayad, Messad, Zenia, Naim and Hamdi [77] found that *Campylobacter* was present in 55% of 100 turkey neck skins sampled from three traditional and one modern abattoir located in the country’s middle regions, Algiers, Bouïra, and Boumèrdes. The abattoirs were characterised by a lack of disinfection protocols and sterilisation of equipment, and by the use of dirty uniforms. These are important factors that enhance the spread of pathogens. However, Laidouci, Mouffok and Hellal [78] found a lower presence of *Campylobacter* (17.9%) in 346 chicken neck and giblets sourced from Algeria.

In Egypt, 24% of 104 chicken carcasses from two slaughterhouses in the Assiut Governorate south of Cairo contained *Campylobacter* [52]. An analysis of 150 raw milk, kareish cheese, and yoghurt samples from September 2014 to February 2015 found that 24.6% tested positive for *Campylobacter* spp. [79]. All the samples tested positive for *C. jejuni* and negative for *C. coli*. The samples were obtained from the local market and street vendors in the city of Qena in southern Egypt. After the samples were cultured, mPCR was used to confirm the isolates. The presence of *Campylobacter* in raw milk was attributed to contamination during on-farm milking processes or to poor post-milking storage and handling conditions. Unhygienic methods used in the preparation, processing, handling, and sales of kareish cheese accounted for it having the highest presence of *Campylobacter* among the products tested. In a study conducted across the governorates of Cairo, Minya, Qalyubia, and Fayoum in Egypt, Omara, Fadaly and Barakat [55] found that *Campylobacter* was present in 12.8% of raw chicken intestines, 9.6% of 680 raw chickens, 1.2% of 344 yoghurt samples, 2.0% of 457 raw milk samples, and 1.7% of 288 kareish cheese samples. The contamination was attributed to unsanitary food production and storage practices. Further, Awadallah, Ahmed, El-Gedawy and Saad [56] found that *Campylobacter* was present in 47.5%, 25.9%, and 21.6% of chicken skins, chicken thighs, and chicken breasts, respectively. These products were sourced from a slaughterhouse in Zagazig, a city in the eastern part of the Nile Delta—the authors found that refrigeration and particularly freezing reduced the counts of viable *Campylobacter* cells. In contrast, in Abu Homos in northern Egypt, a study detected *Campylobacter* in only 2.64% of 227 milk and milk product samples [80]. Using culture methods and mPCR assays, raw milk and fresh domiati cheese (a moderately slated enzyme-coagulated soft cheese) samples tested positive for *Campylobacter.* The remaining milk products, laban rayeb (traditional low-fat fermented milk), stored domiati cheese (a highly salted enzyme-coagulated soft cheese), zabady (Egyptian yoghurt), ras cheese (a hard cheese variety), and kareish cheese (an acid-coagulated soft cheese) were all negative for *Campylobacter*. The products that tested positive were contaminated with *C. jejuni*, which had survived preservation methods better than expected, implying that the pathogen has the ability to develop adaptive strategies to aid survival in food preservation conditions.

In Morocco, *Campylobacter* was detected in 62% of 50 retail poultry samples sourced from the Oujda area in the eastern part of the country [81]. Similar to other countries, the high level of contamination was attributed to poultry being the primary host of *Campylobacter* and cross-contamination occurring at unhygienic slaughter and retail points. The authors further identified the antibacterial effects of common condiments, finding that that 1% v/v lemon juice and vinegar and 2% v/v cinnamon and sodium chloride had high inhibitory effects. However, onion, ginger, black pepper, cumin, parsley, garlic, and saffron had minimal or no effect.

## 4. Antimicrobial Resistance (AMR) Patterns of *Campylobacter* in Africa

According to the World Health Organisation [22], surveillance of AMR in *Campylobacter* has identified important levels of resistance to erythromycin and fluoroquinolones in many parts of the world, which appears to be associated with the use of these drugs in poultry and livestock production systems. Some epidemiological studies in humans and animals have established a relationship between antibiotic use and antimicrobial resistance [82,83,84]. Campylobacteriosis is typically a self-limiting disease, which does not usually require antibiotic treatment; however, in some cases, antibiotics may be administered. Fluoroquinolones and macrolides such as ciprofloxacin and erythromycin, respectively, are recommended for the treatment of *Campylobacter* infections in humans [24,85]. However, given their abuse and misuse, resistance to these drugs has emerged. Resistance to macrolides such as erythromycin and azithromycin is primarily attributed to mutations in the m23SRNA gene and decreased permeability of *Campylobacter* cell walls, while resistance to fluoroquinolone is typically moderated by mutation of DNA gyrase and efflux through the outer and inner membranes [86].

In this review we will focus on the resistance patterns of *Campylobacter* spp. from African environments especially to fluoroquinolones and macrolides as these antimicrobials are the most commonly used for the treatment of *Campylobacter* infections in humans. Table 3 provides a summary of resistance trends.

### 4.1. East Africa

In Jimma, Ethiopia, erythromycin, ciprofloxacin, gentamicin, and nalidixic acid were reportedly effective for more than 80% of *Campylobacter* species isolated from diarrhoeal children using the Kirby–Bauer disc diffusion method. However, 39.5% of the isolates were resistant to tetracycline and 31.6% to chloramphenicol, while resistance to ciprofloxacin and erythromycin was 12.0% [5]. Multidrug resistance was also observed in 78.9% of the isolates. The high resistance to tetracycline, for example, is attributed to its wide availability without prescription; hence, overuse may have resulted in selective pressure on bacteria, thereby making them resistant.

In Kenya, 74% of *C. jejuni* isolates from backyard chickens in Thika, north east of the capital Nairobi, showed resistance to nalidixic acid, while 71% were resistant to each of tetracycline and ciprofloxacin [87]. In that study in Kenya [86], the uncontrolled use and ease of access to antibiotics by small-scale farmers were suggested as reasons for the high resistance rates recorded. In Mozambique, a resistance rate of 11% was reported for ciprofloxacin and nalidixic acid in stool samples of diarrhoeal children at the Manhica Hospital [36]. Such relatively low rates might be expected as the use of fluoroquinolones is not recommended in young children [36].

In Tanzania, *Campylobacter* isolates from milk and beef collected from the Eastern (Morogoro), Northern (Arusha) and South Western (Iringa) parts of the country showed resistance to ciprofloxacin ranged from 9.3% to 11.8%, while the range for erythromycin resistance was between 53.7% and 70.6% [65]. The authors also noted that, in Tanzania, the macrolide, Tylosin, was extensively used for treating respiratory infections like *Mycoplasma* in cattle and its usage for treatment and growth promotion might have contributed to the selection of resistant strains to erythromycin. Furthermore, isolates from free-range ducks in the same city, were susceptible to nitrofurantoin and amikacin, while they showed 74% resistance to tetracycline [66].

### 4.2. West Africa

In Burkina Faso, isolates from human stools showed 13.8% resistance to ciprofloxacin, 10.3% to tetracycline, and 10.3% to erythromycin [41]. A study in Abidjan, Ivory Coast Goualie, Essoh, Elise Solange, Natalie, Souleymane, Lamine Sebastien and Mireille [6], reported 79.5%, 38.5%, 17.9%, and 10.3% resistance of poultry *C. jejuni* isolates to nalidixic acid, ciprofloxacin, amoxicillin, and erythromycin, respectively. *C. coli*, on the other hand, showed resistance rates of 78.4%, 43.2%, 13.5%, and 8.1% to nalidixic acid, ciprofloxacin, amoxicillin, and erythromycin, respectively. The authors noted that the unrestricted use of antibiotics for treatment and as growth promoters contributed to the resistance.

In neighbouring Ghana, a resistance of 92.3–100% was reported for erythromycin, 92.3–93.3% for tetracycline, and 0% for imipenem for isolates from patients in Kumasi [42]. Erythromycin resistance arises as a result of prolonged exposure and evidently, as noted by the authors, erythromycin had been on the Ghanaian market for a long time and was subjected to abuse and misuse. Additionally, the isolates from carcasses and faeces of poultry in Kumasi were all completely sensitive to imipenem. However, the resistance to quinolones ranged from 41–86%, 100% to erythromycin and 97–100% to tetracycline [71]. Furthermore, the isolates from livestock showed 7–69% resistance to quinolones, 97–100% to erythromycin, and 48–94% to tetracycline. There was 0% resistance to imipenem [72]. In Sokoto, (North Western Nigeria), the isolates from cloacal swabs of poultry showed resistance to most of the antibiotics except chloramphenicol. Multidrug-resistant traits were found in 82.1% of the isolates. Resistance to ciprofloxacin was 19.6%, ampicillin 32.1%, tetracycline 24.1%, and erythromycin 11.6% [88]. Due to the low levels of resistance recorded for erythromycin, it was recommended for treatment. The zero resistance to chloramphenicol was because it had been banned for use in both humans and animals [88]. Additionally, in Ilorin in the middle belt of Nigeria, Samuel, Aboderin, Akanbi II, Adegboro, Smith and Coker [23] reported resistance of human isolates to nalidixic acid (24%) and tetracycline (12%) but the isolates showed no resistance to erythromycin and ciprofloxacin. Besides, the isolates from diarrhoeal stools of children below age 3 years in Osogbo, South Western Nigeria [25], were reported to be sensitive to erythromycin, tetracycline, and ciprofloxacin, and were hence recommended for treatment of *Campylobacter* infection. The former study pointed out that all isolates were not sensitive to cotrimoxazole [25].

### 4.3. Southern Africa

A study in Durban, South Africa, reported 68% and 64% resistance to tetracycline in the isolates of *Campylobacter* from chicken and humans, respectively [89]. Also in Durban, Shobo, Bester, Baijnath, Somboro, Peer and Essack [90], reported that 31.5%, 50%, and 25.9% of *C. jejuni* isolates from humans were resistant to erythromycin, azithromycin, and tetracycline, respectively; while 38.9%, 77%, and 55.6% of *C. coli* strains showed resistance to erythromycin, azithromycin, and erythromycin, respectively. In *Campylobac*ter, resistance to tetracycline is primarily mediated by a ribosomal protection protein (*tet*O) that is transferred as a plasmid-encoded gene or in the chromosome where it is not self-mobile [90].

Production systems and bird type also have an influence on isolation rates and resistance. In the Kwazulu Natal province of South Africa, it was established that the isolates from commercially raised, i.e., free range and industrial chickens were highly resistant to tetracycline (98.9–100%). Resistance to gentamicin and streptomycin was 1.6% and 11.5% respectively in the commercial free-range broiler, 1.7% and 16.4% in industrially raised broilers, and 12.9 and 40% respectively in industrially raised layers [92]. Different production systems use antibiotics differently and this accounted for the varied resistance profiles. In 2012, a study found higher resistance to ciprofloxacin in cattle than broilers in the Venda Region in the north of South Africa [91]. In chicken, *C. jejuni* showed a resistance of 29% to ciprofloxacin while in cattle the resistance was 33.3%. However, resistance to erythromycin was higher at 56.7% and 42.9% in chicken and cattle respectively. Resistance to ciprofloxacin among chicken was due to the use of sarafloxacin and enrofloxacin on poultry farms. Additionally, Bester and Essack [28] found varying resistances to common antibiotics used in broilers and layers in Kwazulu Natal. For instance, resistance to tetracycline was 98.2% and 100% for broilers and layers respectively. Resistance to gentamicin was equally high at 98% for broilers and 81% for layers. Thirdly, multi-resistance was found in 23% and 43% of the isolates from broilers and layers, respectively.

### 4.4. North Africa

In Algeria, isolated strains from turkeys showed resistance to tetracycline at 81.3%, ciprofloxacin at 75.0%, and erythromycin at 25.0%. 96.9% of the isolates were multidrug resistant and 18 drug resistance patterns were identified [77]. The authors in the previous study argued that the prudent use of fluoroquinolones contributed to the high resistance rates recorded in ciprofloxacin. The continuous use of fluoroquinolones in poultry production selects for fluoroquinolone-resistant mutants which leads to the emergence of resistant *Campylobacter*. The selection pressure presented by different antibiotics accounted for the varying rate of resistance and multidrug resistance trends recorded. Meryem, Zehor, Fares, Sadjia and Amina [29], also discovered that the isolates from livestock showed resistance of 91.6% to ciprofloxacin, 88.54% to erythromycin, and 44.7% to tetracycline in the middle area of Algeria. Resistance to ciprofloxacin and erythromycin were 91.6% and 88.54% respectively while that to tetracycline was 44.7%.

Reports from Egypt indicated that *Campylobacter* isolates from poultry showed a 58.82% resistance to erythromycin and tetracycline; whereas isolates from humans showed a resistance of 75% to tetracycline and 62.5% to erythromycin [57]. The inexpensive cost and broad-spectrum properties of tetracycline resulted in the widespread use in both humans and animals and consequently, the selective pressure led to the emergence of resistant genes. In addition, *C. jejuni* and *C. coli* isolates from diarrhoeal patients in Cairo showed 40% and 24% resistance to nalidixic acid, 6% and 24% to tetracycline and 9% and 10% resistance to erythromycin respectively [59].

## 5. Management Strategies: Opportunities and Challenges

A holistic, multiple intervention One Health approach is required to better understand, prevent, and control *Campylobacter* and its related infections. One Health recognizes that the health of people is connected to the health of animals and the environment. It is a collaborative, multisectoral, and transdisciplinary approach—working at the local, regional, national, and global levels—with the goal of achieving optimal health outcomes recognising the interconnection between people, animals, plants, and their shared environment (readers unfamiliar with the One Health concept could consult the following CDC website for an introduction: https://www.cdc.gov/onehealth/index.html). In the African context, management of *Campylobacter* can be dealt with at the domestic, farm, processing, and policy levels.

At the domestic setting level, several studies across Africa indicated a significant association between *Campylobacter* enteritis and contact with both animals [32,41,42,93,94] and diarrhoea sick persons [32]. This creates a public health concern since agriculture, particularly the livestock sector, is a major source of livelihood in Africa. In rural areas where sometimes access to adequate medical intervention is unavailable the situation becomes more critical. Furthermore, the communal living culture in Africa tends to have healthy family members taking care of sick ones, and those with diarrhoea are no exceptions from receiving family care. It is important to minimise human-animal contact, practice personal and environmental hygiene, and seek proper medical care for sick persons in a household in order to minimise risks of transmission.

Animal production and management systems play an important part in *Campylobacter* control and must be carefully considered. Studies in Europe estimated that a three log unit reduction of *Campylobacter* load in the ceca of poultry would result to a more than 90% reduction of human infections attributed to poultry meat consumption [22]. Additionally, the animal production system influences antimicrobial usage and this has implications for antibiotic resistance. According to Jonker and Picard [84], in intensive poultry and pig rearing systems the use of oral antibiotics is essential to maintain health hence there is a high risk for the *Campylobacter* in the intestinal tract of food animals to develop resistance to commonly used antibiotics. In poultry flocks, the risk factors for *Campylobacter* introduction include the partial depopulation of flocks at several occasions, the increased age of sale, inadequate washing and disinfection of poultry houses, and poor levels of biosecurity measures such as absence of a footbath at the entrance and poor rodent control [95]. Treatment of drinking water and litter, rodent control on farms, and effective biosecurity are important management interventions to be employed on the farm [96]. Applying biosecurity interventions at poultry production sites has resulted in different levels of success in different countries [6,14,97]. Such variation may be attributed to differences in the *Campylobacter* loads in the poultry chain and environment. Hence, the effectiveness of biosecurity-related interventions in primary production should be based on a good understanding of the regional risk factors at the farm level.

In addition to primary interventions at the farm level, there is a need to apply interventions at the slaughter and processing levels in order to reduce the contamination of poultry meat meant for human consumption. Freezing of contaminated poultry carcasses is a reliable intervention to achieve a 1 to 2-log reduction of *Campylobacter* counts. The compulsory freezing of processed broilers from *Campylobacter*-positive broiler flocks in Iceland resulted in a substantially reduced number of human cases of *Campylobacter* enteritis and is currently being used on a voluntary basis in Norway, Sweden, and Denmark [14,22]. However, in Africa, freezing might not be a feasible option in many countries due to the added cost of energy used for freezing operations as well as challenges in keeping a sustainable cold chain in several countries with limited infrastructure. Many consumers across the African continent prefer to buy fresh poultry meat with no change in product quality [24].

Chemical decontamination can also be an effective intervention for reducing *Campylobacter* load on food animal carcasses, and the feasibility of this option could be appealing in the African context. Chlorine, chlorine dioxide, acidified sodium chlorite, trisodium phosphate, and peroxyacid are typically used in poultry processing in the United States and Australia either as sprays or washes for online reprocessing or added to the chill water tank [7,9]. However, pathogens decontamination using chemicals and its application to poultry carcasses should be well regulated and monitored, in order to avoid excessive use. Also the use of chemical decontamination should not be considered as a replacement, rather complimentary, to good processing practices in poultry abattoirs [14].

The lack of national surveillance data hinders the adequate assessment of the public health impact and burden of disease [7,22]. Most African countries have no national surveillance programs on *Campylobacter*. Therefore, getting the accurate burden of the disease is difficult [24]. Adopting multisector collaborations would help strengthen the disease surveillance system, enhance laboratory capacity, and support the implementation of prevention and control strategies; it would further enhance public health and veterinary laboratories and create intersectorial linkages to tackle zoonotic diseases [97]. In spite of technological advances, laboratories in many African countries still face challenges with isolation and identification of *Campylobacter* in food and clinical samples. Inadequately trained personnel, poor laboratory infrastructure, low funding for research on foodborne pathogens like *Campylobacter* are also major limiting factors in many countries across Africa.

## 6. Conclusions

Zoonotic pathogens such as *Campylobacter* cause disease and death, resulting in enormous social and economic losses. Significant gaps exist in the epidemiological understanding of *Campylobacter* in Africa, and its role as a diarrhoeal agent needs more attention. The present review highlights the variability in the reported occurrence of *Campylobacter* in humans and animal food sources across different countries and regions in Africa. *Campylobacter* infection is particularly prevalent in the paediatric population and has been isolated from farm animals, particularly poultry, and foods of animal origin. The reported prevalence of *Campylobacter* in children under the age of five years ranges from 2% in Sudan to 21% in South Africa. To better understand the magnitude of the campylobacteriosis burden, future research is required to evaluate the under-reporting of diarrhoea incidents at national and continental levels; this cannot be achieved without enhancing local capacities for disease surveillance and monitoring.

A holistic, multiple intervention One Health approach is required to better understand, prevent, and control *Campylobacter* in the African context. The management of the foodborne transmission of *Campylobacter* can be dealt with at the domestic, farm, processing, and policy levels. In poultry, the present review points that the prevalence ranges from 14.4% in Ghana to 96% in Algeria. This review also highlights the alarming trend across several countries in Africa of increased *Campylobacter* resistance to clinically important antimicrobials, such as ciprofloxacin and erythromycin, in humans and food animals. 

In our opinion, and based on the evidence gathered in this review, we believe that *Campylobacter* infection is predicted to emerge further as a serious challenge that African nations will face in the near future. Children in low-resource settings countries across Africa will be suffering the most, given the high burden of infection, combined with the growing trend in *Campylobacter* resistance to clinically relevant antimicrobial. We still know little about from what, how, and where children contract infection. What role do domestic animals, which are known reservoirs of *Campylobacter*, play in transmission? Does infection result from fecal contamination in the environment and how long does *Campylobacter* survive in the environment? Although exposure to poultry may be important, identified determinants are varied across different regions in Africa. Given the paucity of current data, further research on *Campylobacter* in Africa is warranted. Epidemiological studies on risk factors and exposure routes would assist in devising appropriate interventions and strategies. However, it is important to prioritise the reduction of risk factors and exposure routes in all settings as a first step in management.

## Figures and Tables

**Table 1:** Prevalence Rates of *Campylobacter* in Humans in Some African Countries.

Region/Country	Population	Sample Size	Prevalence [%]	Genus/Species	Detection Procedure	Reference
**EAST AFRICA**						
Ethiopia						
Jimma/South Western	Diarrhoeal children under 5 years	227	16.7	*Campylobacter*	Cultural	[5]
			71.1	*C. jejuni*		
			21.1	*C. coli*		
			7.9	*C. lari*		
Gondar/North Western	Diarrhoeal children under age 5	285	15.4	*Campylobacter*	Cultural	[31]
Kola Diba/North Western	Children under age 15	153	10.5	*Campylobacter*	Cultural	[32]
Kenya/South Western	Diarrhoeal children	156	5.8	*Campylobacter*	Cultural	[33]
	Controls	156	1%			
Madagascar	Diarrhoeal children 0–60 months old	2196	9.7	*Campylobacter*	Cultural	[34]
Malawi/ Balantyre/ Southern	Diarrhoeal children	1941	21	*Campylobacter*	PCR	[35]
	Non-diarrhoeal Children	507	14.1	*Campylobacter*		
Mozambique	Diarrhoeal children	529	1.7	*Campylobacter*	Cultural	[36]
Tanzania						
Morogoro/Eastern	Patients	1195	11.5	*Campylobacter*	Cultural, MALDI-TOF	[37]
			84.1	*C. jejuni*		
			15.9	*C. coli*		
Mwanza/Northern	Diarrhoeal children	300	9.7	*Campylobacter*	Cultural	[38]
Morogoro/Eastern	Diarrhoeal patients	632	9.3	*Campylobacter*	Cultural with Skirrow’s protocol and PCR	[39]
			96.6	*C. jejuni*		
			3.4	*C. coli*		
Uganda, Kampala	Diarrhoeal children	226	9.3	*Campylobacter*	Cultural	[27]
			80.9	*C. jejuni*		
			9.5	*C. lari*		
			4.5	*C. coli*		
			4.5	*C. lari/C. jejuni*		
**CENTRAL AFRICA**						
Angola, Luanda	Diarrhoeal children under 5	194	15	*Campylobacter*	Multiplex PCR	[40]
		98	23			
	Non diarrhoeal under 5	96	6			
**WEST AFRICA**						
Burkina Faso, Ouagadougou	Enteritis patients	1246	2.3	*Campylobacter*	Cultural	[41]
			51.8	*C. jejuni*		
			13.8	*C. coli*		
			3.5	*C. upsaliensis*		
Ghana Kumasi	Diarrhoeal and urinary tract infection patients	202	17.3	*Campylobacter*	Cultural	[42]
			40	*C. jejuni*		
			2.8	*C. jejuni subs doylei*		
			37	*C. coli*		
			20	*C.lari*		
Liberia Urban coastal Rural forest	Children 6–59 months	859		*Campylobacter*	Cultural with Skirrow’s protocol	[43]
		341 urban	44.9			
		518 rural	28			
Nigeria						
Sokoto/North Western	Diarrhoeal patients	292	55	*Campylobacter*	Cultural	[44]
Sokoto/North Western	Pregnant women	23	70	*Campylobacter*	Cultural	[45]
Enugu/South Eastern	Diarrhoeal children	514	8.3	*Campylobacter*	Cultural	[46]
			93	*C. jejuni*		
Ilorin/Middle Belt	Diarrhoeal children	306	8.2	*Campylobacter*	Cultural with Butzler type media	[23]
			56	*C. jejuni*		
			44	*C. coli*		
**SOUTHERN AFRICA**						
Venda/Northern	Human stools	322	10.2	*C. jejuni*	PCR	[47]
			6.5	*C. coli*		
			3.1	*C.conscisus*		
Vhembe/North most	Diarrhoeal stools	565	20.3	*Campylobacter*	Cultural with Cape Town Protocol, PCR	[48]
Cape town/ Coastal	Diarrhoeal stools	5443	40	*C. jejuni*	Cultural with Cape Town Protocol	[49]
			24.6	*C. concius*		
			23.6	*C. upsaliensis*		
Limpopo, North Eastern	HIV individuals	60	20	*Campylobacter*	Cultural	[50]
Durban/South Eastern	Diarrhoeal children under 5	126	21	*Campylobacter*	Cultural	[51]
**NORTH AFRICA**						
Egypt						
Assiut/South of Cairo	Human	80	27.5	*Campylobacter*	Cultural and Molecular	[52]
Abu Homos/ Northern	Children	6562	9.37	*Campylobacter*	Cultural	[53]
North of Cairo	Rural children	106	12.3	*C. jejuni*	Cultural	[54]
			2.8	*C. coli*		
South, South East and North of Cairo	Occupational workers	274	8.4%	*Campylobacter*	Biochemical and Molecular	[55]
Zagazig/East Nile Delta	Human	110	2.7	*Campylobacter*	Molecular	[56]
			5.2	*C. jejuni*		
			3.2	*C. coli*		
Giza/Central	Human stools	48	16.66	*Campylobacter*	Cultural	[57]
Abu Homos/Northern	Diarrhoeal children under three years	396	10.5	*Campylobacter*	Cultural	[58]
Cairo	Human	869	16.8	*Campylobacter*	Cultural	[59]
Sudan (Khartoum)	Diarrhoeal children	437	2	*Campylobacter*	Cultural, PCR	[60]

**Table 2:** Occurrence of *Campylobacter* in Foods of Animal Origin.

Region/Country	Product	Sample Size	Percentage Positive	Genus/Species	Detection Procedure	Reference
EAST AFRICA						
Ethiopia/South East Addis Ababa	Sheep and goat carcass	398	10.1	*Campylobacter*	Cultural	[63]
			72.5	*C. jejuni*		
			27.5	*C.coli*		
Kenya/Nairobi	Chicken	100	77	*Campylobacter*	Cultural	[64]
	Beef	50	2			
Tanzania						
Northern, South Western, Eastern	Beef carcass	253	9.5	*Campylobacter*	mPCR	[65]
	Raw milk	284	13.4			
Morogoro/Eastern	Duck intestines	90	80	*Campylobacter*	Cultural with Skirrow’s protocol	[66]
Morogo/Eastern	Cattle carcass	107	9.3	*Campylobacter*	Cultural with Skirrow protocol	[67]
**CENTRAL AFRICA**						
Cameroon/Yaounde	Chicken	150	90	*Campylobacter*	Cultural	[68]
Congo DR/Lubumbashi	Goat meat	177	41.2	*Campylobacter*	PCR	[69]
	Goat stomach	86	37.2			
	Ready to eat goat skewer	139	23.7			
**WEST AFRICA**						
Burkina Faso/Ouagadougou	Chicken carcass	20	50	*Campylobacter*	Cultural	[70]
Ghana						
Kumasi	Poultry carcass	132	21.9	*Campylobacter*	Cultural	[71]
			79	*C. jejuni*		
			14	*C. coli*		
			4	*C. jejuni subs doylei*		
			3	*C. lari*		
Kumasi	Cattle carcass	110	34.5	*Campylobacter*	Cultural, mPCR	[72]
	Goat carcass	134	23.9			
	Sheep carcass	117	35.9			
	Pig carcass	102	36.3			
Nigeria						
Sokoto/North Western	Raw milk	146	4.8	*Campylobacter*	Cultural	[73]
			100	*C. jejuni*		
Sokoto/North western	Chicken	681	81.9	*Campylobacter*	Cultural	[74]
			60.9	*C. jejuni*		
			39.1	*C.coli*		
Senegal/Dakar	Chicken	300	56	*Campylobacter*	Cultural	[75]
**SOUTHERN AFRICA**						
South Africa Gauteng/North	Chicken carcass	99	32.3	*Campylobacter*	Cultural	[76]
**NORTH AFRICA**						
Algeria						
Middle area	Turkey neck skin	100	55	*Campylobacter*	Cultural	[77]
Middle area; Algiers, Bouira, Boumerdes	Chicken neck, giblets	346	17.9	*Campylobacter*	Cultural with Butzler medium and Skirrow’s protocol	[78]
Egypt						
Assiut/South of Cairo	Chicken	104	24	*Campylobacter*	Cultural	[52]
Qena city/Southern	Milk, cheese, yogurt	150	24.6	*Campylobacter*	Cultural, PCR	[79]
Cairo, Minya, Qalubiya and Fayoum	Yogurt	344	1.2	*Campylobacter*	Cultural	[55]
	Raw milk	457	2.0			
	Cheese	288	1.7			
	Chicken intestine	211	12.8			
	Chicken meat	9.6	680			
Zagazig/Eastern Nile Delta	Chicken breast	64	47.5	*Campylobacter*	Cultural	[56]
	Chicken thighs	64	25.9			
	Chicken skin	64	21.6			
Abou Homos/Northern	Milk, milk products	227	2.64	*C. jejuni*	mPCR	[80]
Morocco/ Oujda/Eastern	Poultry carcass	50	62	*Campylobacter*	Cultural, Hippurate hydrolysis	[81]
			90	*C. jejuni*		
			10	*C. coli*		

**Table 3:** Antibiotic Resistance Trends of *Campylobacter* in Some African Countries.

Region/Country	Source	Species	Antibiotic	Resistance %	Method Used	Reference
**EAST AFRICA**						
Ethiopia/South Western	Humans, diarrhoeal children	*Campylobacter*	Tetracycline	39.5	Kirby-Bauer Disk Diffusion	[5]
			Chloramphenicol	31.6		
Kenya, North eastern Nairobi	Backyard chicken	*C. jejuni*	Tetracycline	71	PCR	[87]
			Ciprofloxacin	71		
			Nalidixic acid	77.4		
			Chloramphenicol	25.8		
Mozambique	Diarrhoeal children	*Campylobacter*	Tetracycline	22	Disk Diffusion	[36]
			Ciprofloxacin	11		
			Chloramphenicol	11		
			Nalidixic acid	11		
Tanzania						
Morogo/Eastern	Free range ducks	*Campylobacter*	Erythromycin	20–50	Disk Diffusion	[66]
Iringa/North, Morogoro/Eastern Arusha/South West	Milk, beef	*Campylobacter*	Ciprofloxacin	9.3	Disk Diffusion	[65]
			Erythromycin	53.7		
			Tetracycline	18.5		
			Azithromycin	42.6		
			Nalidixic acid	64.8		
			Chloramphenicol	13		
			Ciprofloxacin	11.8		
			Erythromycin	70.6	Broth micro-dilution	
			Tetracycline	17.7		
Morogoro/Eastern	Humans	*Campylobacter*	Ciprofloxacin	22.1	Disk Diffusion	[37]
Uganda/ Kampala	Humans	*Campylobacter*	Ciprofloxacin	5	Disk Diffusion	[27]
**WEST AFRICA**						
Burkina Faso/ Ouagadougou	Humans	*Campylobacter*	Ciprofloxacin	13.8	Disk Diffusion	[41]
			Tetracycline	10.3		
			Erythromycin	10.3		
			Nalidixic acid	34.5		
Ivory Coast/Abidjan	Chicken	*C. jejuni*	Ciprofloxacin	38.5	Disk Diffusion	[6]
			Erythromycin	10.3		
			Nalidixic acid	79.5		
		*C. coli*	Ciprofloxacin	43.2	Disk Diffusion	
			Erythromycin	8.1		
			Nalidixic acid	78.4		
Ghana						
Kumasi/South	Humans	*Campylobacter*	Erythromycin	92.3–100	Disk Diffusion	[42]
			Tetracycline	92.3–93.3		
Kumasi/South	Poultry carcass and faeces	*Campylobacter*	Quinolones	41–86	Kirby-Bauer Disk Diffusion	[71]
			Erythromycin	100		
			Tetracycline	97–100		
Kumasi/South	Livestock	*Campylobacter*	Quinolones	7–69	Kirby-Bauer Disk Diffusion	[72]
			Erythromycin	97–100		
			Tetracycline	48–94		
Nigeria						
Sokoto/North Western	Poultry cloacal swabs	*Campylobacter*	Erythromycin	11.6	Disk Diffusion	[88]
Osogbo/South Western	Humans	*Campylobacter coli*	Ciprofloxacin	0	Kirby-Bauer Disk Diffusion	[25]
			Nalidixic acid	66		
			Erythromycin	0		
			Tetracycline	0		
	
Ilorin/Middle belt	Humans	*Campylobacter*	Ciprofloxacin	0	Kirby-Bauer Disk Diffusion	[23]
			Erythromycin	0		
			Nalidixic acid	24		
			Tetracycline	12		
**SOUTHERN AFRICA**						
Durban/South Eastern	Humans	*Campylobacter*	Tetracycline	64	PCR	[89]
	Chicken			68		
Durban/South Eastern	Humans	*C. jejuni*	Erythromycin	31.5	Broth microdilution	[90]
			Azithromycin	50		
		*C. coli*	Erythromycin	38.9		
			Azithromycin	77		
Vende/ Northern	Cattle	*C. jejuni*	Ciprofloxacin	33.3	Disk diffusion	[91]
			Erythromycin	42.9		
			Nalidixic acid	26.2		
			Tetracycline	31		
	Chicken		Ciprofloxacin	29		
			Erythromycin	56.7		
			Nalidixic acid	47.8		
			Tetracycline	33.3		
	Cattle	*C. coli*	Ciprofloxacin	56.3		
			Erythromycin	6.8		
			Nalidixic acid	37.5		
			Tetracycline	62.5		
	Chicken		Ciprofloxacin	37.5		
			Erythromycin	43.8		
			Nalidixic acid	31.3		
			Tetracycline	43.8		
Kwazulu Natal	Commercial free range broilers	*Campylobacter*	Tetracycline	100	Agar Dilution	[92]
			Ciprofloxacin	95.4		
			Erythromycin	87.9		
	Rural birds		Tetracycline	21.6		
			Ciprofloxacin	7.9		
			Erythromycin	0		
	Industrial broiler		Tetracycline	98.9		
			Ciprofloxacin	15.9		
			Erythromycin	47.6		
	Industrial layer		Tetracycline	100		
			Ciprofloxacin	17.7		
			Erythromycin	43.7		
Kwazulu Natal	Broiler	*Campylobacter*	Tetracycline	98.2	Agar Dilution	[28]
			Erythromycin	50		
			Ciprofloxacin	8.9		
			Nalidixic acid	35.7		
	Layer		Tetracycline	100		
			Erythromycin	42.9		
			Ciprofloxacin	23.8		
			Nalidixic acid	52.4		
**NORTH AFRICA**						
Algeria						
Bouira/ Middle area	Turkey carcasses	*Campylobacter*	Tetracycline	81.3	Disk Diffusion	[77]
			Ciprofloxacin	75		
			Erythromycin	25		
			Nalidixic acid	87.5		
Middle area/Algiers, Bouira &, Boumerdes	Sheep, calves broiler carcasses	*Campylobacter*	Ciprofloxacin	91.6	Disk Diffusion	[29]
			Erythromycin	88.5		
			Tetracycline	44.7		
			Nalidixic acid	96.8		
Egypt						
Giza	Poultry	*Campylobacter*	Erythromycin	58.8	Disk Diffusion	[57]
			Tetracycline	58.8		
			Chloramphenicol	64.7		
	Human		Erythromycin	50		
			Tetracycline	75		
			Chloramphenicol	50		
Cairo	Human	*C. jejuni*	Nalidixic acid	40	Disk diffusion	[59]
			Tetracycline	6		
			Erythromycin	9		
			Chloramphenicol	3		
		*C. coli*	Nalidixic acid	24		
			Tetracycline	24		
			Erythromycin	10		
			Chloramphenicol	0		
